# Towards a Better Detection of Horizontally Transferred Genes by Combining Unusual Properties Effectively

**DOI:** 10.1371/journal.pone.0043126

**Published:** 2012-08-14

**Authors:** Dapeng Xiong, Fen Xiao, Li Liu, Kai Hu, Yanping Tan, Shunmin He, Xieping Gao

**Affiliations:** 1 Key Laboratory of Intelligent Computing & Information Processing of Ministry of Education, Xiangtan University, Xiangtan, Hunan, People’s Republic of China; 2 Key Laboratory of Zoological Systematics and Evolution, Institute of Zoology, Chinese Academy of Sciences, Beijing, People’s Republic of China; Aligarh Muslim University, India

## Abstract

**Background:**

Horizontal gene transfer (HGT) is one of the major mechanisms contributing to microbial genome diversification. A number of computational methods for finding horizontally transferred genes have been proposed in the past decades; however none of them has provided a reliable detector yet. In existing parametric approaches, only one single compositional property can participate in the detection process, or the results obtained through each single property are just simply combined. It’s known that different properties may mean different information, so the single property can’t sufficiently contain the information encoded by gene sequences. In addition, the class imbalance problem in the datasets, which also results in great errors for the gene detection, hasn’t been considered by the published methods. Here we developed an effective classifier system (Hgtident) that used support vector machine (SVM) by combining unusual properties effectively for HGT detection.

**Results:**

Our approach Hgtident includes the introduction of more representative datasets, optimization of SVM model, feature selection, handling of imbalance problem in the datasets and extensive performance evaluation via systematic cross-validation methods. Through feature selection, we found that JS-DN and JS-CB have higher discriminating power for HGT detection, while GC1–GC3 and k-mer (k = 1, 2, …, 7) make the least contribution. Extensive experiments indicated the new classifier could reduce Mean error dramatically, and also improve Recall by a certain level. For the testing genomes, compared with the existing popular multiple-threshold approach, on average, our Recall and Mean error was respectively improved by 2.81% and reduced by 26.32%, which means that numerous false positives were identified correctly.

**Conclusions:**

Hgtident introduced here is an effective approach for better detecting HGT. Combining multiple features of HGT is also essential for a wider range of HGT events detection.

## Introduction

Horizontal gene transfer (HGT, also called lateral gene transfer) is a transfer of genetic material from one lineage to another and has played a key role in species evolution and microbial genome diversification [1], [2]. Transfers can occur both between closely and distantly related species or strains, and are thought to be frequent events [3]. In addition, horizontal gene transfer has also been proposed to result in the emergence of novel human diseases and poses several risks to humans [4], [5]. As sequence data has accumulated, evidence for rampant HGT has increased dramatically. Thus, detecting HGT has enormous practical significance for providing a better understanding of the impact of HGT on genome evolution and for identifying new drug targets.

At present, there are two primary strategies to detect the genes that have been transferred horizontally: phylogenetic approaches and parametric approaches [6], [7]. Phylogenetic approaches are typically based on the comparative study of numerous genomes to find genes with unusually taxonomic distributions. However, many other phenomena, such as biased mutation rates, gene loss and long branch length attraction etc., also can cause the phylogenetic tree for a gene to differ from that for the species, thus, phylogenetic approaches are time-consuming and insufficiently robust [8], [9].

In contrast, parametric approaches (also called composition-based approaches) are based on a common theory that the unusual characteristics of horizontally transferred genes can distinguish themselves from other genes in genome. This kind of approach is computationally less demanding and can be carried out in each single genome. So far, various parametric approaches have been proposed, but it’s not difficult to find one common drawback that only one single compositional property could be used to identify the transferred genes in each predicting experiment. It’s known that different properties may mean different information, and this limitation also results in great errors for HGT detection. Some combined methods were also proposed by Becq et al. [10] and Azad et al. [11] to resolve this problem, but these methods just only combined the predictive results that obtained through each single property, the essence remained that only one single property was used. Therefore, how to sufficiently extract the information encoded by genes has become an open and challenging issue. In addition, machine learning also was applied widely for HGT detection [12], [13], but the class imbalance problem which can result in poor classification performance with respect to the minority class [14] hasn’t been considered by them.

In light of all the caveats, in this study, we have developed a new strategy (Hgtident) which used support vector machine (SVM) to detect horizontally transferred genes by combining the unusual properties effectively, meanwhile, the class imbalance problem was also considered. The information from combined properties can sufficiently stands for the whole gene sequence. To our knowledge, this is the first use of such integrated strategy to identify horizontally transferred genes. As a result, Hgtident can achieve better performance than the existing methods.

## Materials and Methods

### Datasets

In previously published study, various artificial datasets were put forward [3], [5], [6], [7], [8], [10], [11], [12], [13], and this kind of simulative dataset was composed of donor genes and recipient genome. The task is that of recovering as many as possible of the donor genes. But it’s important to note that, in the evolutionary histories of recipient genome, those genes from a transfer of genetic materials between different species don’t have been considered.

So, in this article, in order to validate the performance of Hgtident in genuine genomes, we chose six common genomes published in more reliable HGT-DB database (http://genomes.urv.cat/HGT-DB/) [15], which was *E. coli* K12, *E. coli* O157 Sakai, *S. enterica* Typhi CT18, *S. enterica* Paratypi ATCC 9150, *C. pneumoniae* CWL029 and *S. agalactiae* 2603, respectively. The horizontally transferred genes and others in genome were respectively regarded as positives and negatives. The results predicted by Hgtident would be compared with that of the existing popular multiple-threshold approach proposed by Azad et al. [11].

### Compositional Features

7 common features were used for HGT detection owing to their representativeness and wider coverage. These features contained Karlin’s dinucleotide [16], Karlin’s codon bias [17], GC1–GC3 [18], k-mer (k = 1, 2, …, 7) [19], 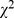 dinucleotide, 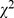 codon bias [20], JS-N, JS-DN and JS-CB [8], which is respectively based on structural, statistical, and Shannon information entropy characteristics. We believe these comprehensive properties can sufficiently express the information encoded by gene sequences. Therefore, we chose them to develop the SVM model.

### Selection of SVM Model

SVM is a supervised machine learning paradigm derived from the statistical learning theory of structural risk minimization principle for solving linear and non-linear classification and regression problem [21]. We chose SVM as our classification paradigm due to its high generalization capability, ability to find global classification solutions [21], and successful application in bioinformatics and other practical domains.

The model selection for SVM involves the selection of a kernel function and its parameters which yield the optimal classification performance for a given dataset [21]. Among the available kernel functions, we chose the most popular and widely used Radial Basis Function (RBF) as the kernel function because of its higher reliability in finding optimal classification solutions in most practical situations [22]. The performance of the classifier at each parameter point (c, g) is evaluated by 5-fold cross-validation on the training dataset. After finding the best parameters, a new SVM model was trained using the complete training dataset at those parameters. Then a separate testing dataset was used to measure the performance of the developed classifier. The C++ interface of libsvm3.1 package [23] was used to develop SVM model. Before training the SVM classifier systems, the complete dataset was scaled into (−1, +1) interval.

### Feature Selection

Selecting the most discriminative set of features would increase the performance, efficiency and comprehensibility of a classifier system by reducing its complexity. In particular, through analyzing the optimal feature subsets for these genomes, we can clearly realize which features make more important contributions to HGT detection. Here genetic algorithm (GA) was chosen as our feature selection paradigm due to its strong random search ability to find the convincingly optimal feature subset. The evaluation from GA aims to one feature subset, not one single feature, and this can guarantee the combination optimization of feature subset [24]. Firstly, generated some feature subsets randomly, then the new feature subsets were obtained through selection, cross and mutation. After many iterations of this ritual, the result would converge to the optimal solution, which corresponded to the optimal feature subset. The 5-fold cross-validation was used to test the generalization ability of feature subsets, the feature subset which obtained optimal classification performance would be considered as optimal feature subset.

The feature selection procedure was carried out based on initial imbalanced dataset. Binary string was chosen to code the feature data of the population, 1 meant that the corresponding feature was selected, and 0 was just the reverse. The chosen fitness function was f(x) = 10000*Recall because we needed to evaluate the Mean error under the situation where the highest Recall was obtained. Each generation contained 100 individuals. And the cross probability, mutation probability and iteration number was set to 0.9, 0.1 and 200, respectively. We employed the classical proportion selection operators, and the optimal two individuals in every generation were directly passed to the next generation.

### Class Imbalance Problem

As is well-known, the horizontally transferred genes are far less than others in each genome, which will inevitably result in the sample imbalance problem. It has been well studied that training a classifier with an imbalance positive and negative dataset in machine learning research would result in poor classification performance with respect to the minority class [14], [25], in this case, it would be with respect to the horizontally transferred gene class. According to previous study, Synthetic Minority Over-sampling Technique (SMOTE) which is independent from the learning algorithm and involves in pre-processing of training data was successfully applied to this kind of problem [26]. It is an over-sampling technique which introduces new synthetic examples in the neighborhood of the existing minority examples. Therefore, SMOTE was chosen to resolve the class imbalance problem in this study.

### Evaluation Criteria

In this research, we used detection rate Recall as our primary evaluation criteria as the same with that in the paper published by Azad et al. [11]. In addition, Mean error was also used as the evaluation criteria to sufficiently evaluate the performance of Hgtident. They are defined as follows,
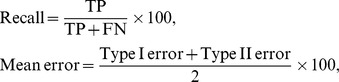
where 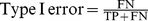, 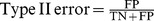, and TP refers to true positives, FN refers to false negatives, TN refers to true negatives and FP refers to false positives.

## Results and Discussion

### Feature Selection Results

As the first experiment, we trained an SVM model with the complete imbalanced dataset to observe the classification performance. The complete dataset was randomly divided into five equally sized partitions and each partition contained the same ratio of positives and negatives. Then four partitions were used together as the training dataset to develop an SVM classifier, the resulted model was tested for its classification performance on the fifth partition. This procedure was repeated five times with different combinations of training and testing dataset, and the results were averaged. Table 1 shows the classification results obtained subjected to all features and optimal feature subset. For each genome, Recall and Mean error were respectively improved and reduced effectively by using the optimal feature subset. In average, Recall was improved by 6.50%, and Mean error was reduced by 4.67%, which showed the optimal feature subset has a significant influence in better classification results. The resulted optimal feature subsets with less number of features not only gave higher classification results, but also immensely reduced computational complexity.

**Table 1:** Comparison of classification results obtained through 5-fold cross validation with respect to different feature subsets selected.

	All features		Optimal feature subset	
Genome	Recall	Meanerror	Recall	Meanerror
*E. coli* K12	80.62	18.67	86.79	13.50
*E. coli* O157 Sakai	76.83	17.97	84.62	14.31
*S. enterica* Typhi CT18	67.32	27.31	77.27	21.92
*S. enterica* Paratypi ATCC 9150	78.34	20.38	83.73	16.05
*C. pneumoniae* CWL029	74.52	19.17	80.66	16.10
*S. agalactiae* 2603	74.73	20.69	78.30	14.27

At the same time, the optimal feature subset for each genome was analyzed as well, and summarized in Table 2. We found the JS-DN and JS-CB appeared in five out of six feature subsets, which indicated these two features have higher discriminating power for HGT detection than the others. Second was Karlin’s codon bias, 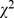 dinucleotide and 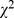 codon bias. In addition, GC1–GC3 and k-mer (k = 1, 2, …, 7) hardly ever appeared in these optimal feature subsets, which also indicated these features make the least contribution to HGT detection. These deductions could also be well achieved from the results obtained through the multiple-threshold approach in Section “Comparison of multiple-threshold approach with Hgtident”.

**Table 2:** The optimal feature subsets for testing genomes.

Feature	A	B	C	D	E	F
Karlin’s dinucleotide		Yes	Yes		Yes	
Karlin’s codon bias	Yes		Yes	Yes		Yes
GC1–GC3				Yes		
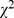 dinucleotide	Yes	Yes		Yes	Yes	
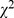 codon bias		Yes	Yes		Yes	Yes
JS-N		Yes	Yes	Yes		
JS-DN	Yes		Yes	Yes	Yes	Yes
JS-CB	Yes	Yes	Yes		Yes	Yes
1-mer	Yes					
2-mer						
3-mer				Yes		
4-mer					Yes	
5-mer			Yes			
6-mer	Yes					
7-mer						Yes

A-F is *E. coli* K12, *E. coli* O157 Sakai, *S. enterica* Typhi CT18, *S. enterica* Paratypi ATCC 9150, *C. pneumoniae* CWL029 and *S. agalactiae* 2603, respectively. “Yes” indicates that the corresponding feature is included in the optimal feature subset.

### Class Imbalance Learning Results

The imbalance learning experiments would be carried out to observe the classification results by 5-fold cross validation. First, an SVM model was trained by applying SMOTE on a training dataset containing four-fifth complete dataset. Then its performance was tested on the remaining imbalanced one-fifth of dataset. This procedure was repeated five times with different combinations of training and testing datasets, finally, the results were averaged. Table 3 presents the classification results through class imbalance learning method with the optimal feature subsets. From these results, we could find that, compared with the preliminary classification results obtained through the imbalanced datasets, the application of SMOTE could improve the Recall and reduce the Mean error effectively. In average, Recall was improved by 6.53%, and Mean error was reduced by 6.02%, which provided a good evidence for us to apply SMOTE in this problem for the development of a better performing classifier with respect to imbalanced positive and negative classes.

**Table 3:** Comparison of classification results obtained through class imbalance learning method with the optimal feature subsets by 5-fold cross validation.

	None (imbalanced dataset)		SMOTE (balanced dataset)	
Genome	Recall	Mean error	Recall	Mean error
*E. coli* K12	86.79	13.50	92.35	7.26
*E. coli* O157 Sakai	84.62	14.31	89.85	9.64
*S. enterica* Typhi CT18	77.27	21.92	86.17	11.36
*S. enterica* Paratypi ATCC 9150	83.73	16.05	91.60	7.64
*C. pneumoniae* CWL029	80.66	16.10	83.51	13.70
*S. agalactiae* 2603	78.30	14.27	87.09	10.42

### Comparison of Multiple-threshold Approach with Hgtident

At present, the multiple-threshold approach proposed by Azad et al. [11] is very popular, because better results can be obtained. Thus the comparison between these two approaches would be carried out to evaluate the performance of Hgtident (Table 4). It’s not difficult to find that, in the multiple-threshold approach, each Recall was obtained at the cost of a higher Mean error, which means that a mass of false positives were produced. The reason is maybe that only one single property can be applied in this approach, however, every one single property can’t sufficiently express the comprehensive information encoded by genes. This information should be expressed sufficiently by different properties based on different directions. Therefore, these seven comprehensive and representative features were applied to this research together. From Table 4, we could clearly observe that Hgtident effectively reduced the Mean error, which also illustrated the correctness of our viewpoint.

**Table 4:** The classification results of the multiple-threshold approach and Hgtident.

	*E. coli* K12		*E. coli* O157 Sakai		*S. enterica* Typhi CT18		*S. enterica* Paratypi ATCC 9150		*C. pneumoniae* CWL029		*S. agalactiae* 2603	
Method	Recall	Mean error	Recall	Mean error	Recall	Mean error	Recall	Mean error	Recall	Mean error	Recall	Mean error
A	81.03	30.54	79.05	22.94	66.80	33.99	89.93	37.32	69.27	37.49	72.35	38.57
B	75.56	28.09	77.51	26.19	71.27	26.99	82.80	22.99	67.43	37.33	76.57	23.12
C	42.44	37.98	47.02	43.10	33.96	47.27	45.65	39.51	40.53	43.15	37.19	46.60
D	64.95	36.56	67.29	29.42	71.79	51.44	78.56	24.23	72.60	44.09	69.50	30.51
E	61.09	38.71	61.67	38.72	61.75	41.90	71.13	24.26	73.34	40.56	80.63	34.73
F	**93.11**	**51.24**	86.03	27.45	**80.22**	**25.39**	82.16	46.98	68.64	37.76	77.50	29.67
G	86.50	30.92	77.00	26.20	78.73	35.25	79.41	24.14	77.05	27.10	67.60	40.06
H	90.03	34.32	**89.12**	**33.50**	70.71	26.89	**90.78**	**43.26**	**79.71**	**28.89**	**80.76**	**35.67**
I	87.13	36.27	47.19	50.51	34.51	58.81	88.93	46.27	46.51	47.38	37.24	52.63
J	43.73	53.22	49.06	51.64	46.27	58.77	46.50	51.87	47.36	50.60	41.15	52.76
K	43.73	53.05	49.06	50.83	41.79	58.49	51.80	51.90	47.36	50.73	41.15	51.32
L	63.73	44.29	50.15	53.19	35.82	58.71	51.80	52.45	53.15	51.19	35.07	49.67
M	43.73	53.34	52.81	49.75	35.82	58.50	46.71	52.00	48.89	51.32	54.13	49.75
N	51.13	53.37	47.53	51.38	44.51	56.80	46.50	51.97	47.36	50.38	46.30	51.69
O	43.73	54.17	49.06	50.92	44.51	56.80	48.83	52.21	49.10	47.41	49.62	51.33
Hgtident	92.35	7.26	89.85	9.64	86.17	11.36	91.60	7.64	83.51	13.70	87.09	10.42

A-O is multiple-threshold approach based on Karlin’s dinucleotide, Karlin’s codon bias, GC1–GC3, 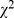 dinucleotide, 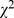 codon bias, JS-N, JS-DN, JS-CB and k-mer (k = 1, 2, …, 7), respectively. The best Recalls and corresponding Mean errors obtained through multiple-threshold approach are depicted in bold face.

**Figure 1:**
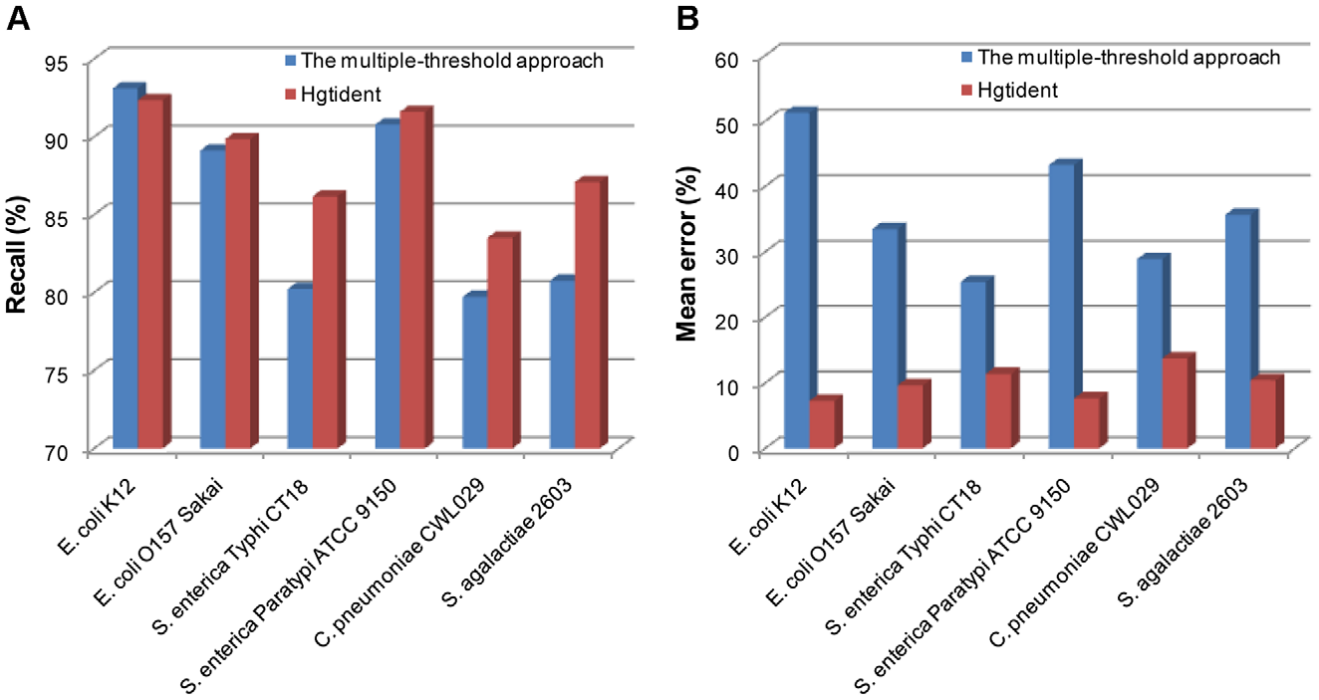
Comparison between Hgtident and the multiple-threshold approach. (A) Comparison of the highest Recalls. (B) Comparison of the Mean errors corresponded to the highest Recalls.

In addition, we respectively chose the highest Recall and the corresponding Mean error obtained through multiple-threshold approach in each genome to compare with our results (Fig. 1). For *E. coli* K12, our Recall was reduced by 0.76%; but for *E. coli* O157 Sakai, *S. enterica* Typhi CT18, *S. enterica* Paratypi ATCC 9150, *C. pneumoniae* CWL029 and *S. agalactiae* 2603, our Recall was respectively improved by 0.73%, 5.95%, 0.82%, 3.80% and 6.33%, as a whole, the mean was 2.81%. In addition, for each genome, our Mean error was reduced dramatically, and the overall mean was 26.32%. Tsirigos et al. [12] and Chen et al. [13] also used SVM to research the prediction of horizontally transferred genes, but they used the simulated datasets, most importantly, only one single property was used in their researches, which also indicated insufficient information encoded by genes was extracted. In addition, surprisingly, none of them have considered a proper class imbalance learning method for classifiers development. Thus, their results were even inferior to that obtained through the multiple-threshold approach. Therefore, we can state that the results reported in our research are much more reliable and better than those results published by other existing approaches.

### Conclusions

In this research, an integrated strategy, which more comprehensively described the biological information encoded by genes, was proposed to identify horizontally transferred genes. Meanwhile, SMOTE was also considered to address the class imbalance problem. Extensive experiments indicated that the extraction of sufficient information can reduce Mean error dramatically, and also improve Recall by a certain level. However, change in gene inventory is a historical process, how to thoroughly extract the useful information encoded by genes still remain a challenging and open issue. Further study is yet needed to decrease the false positives and negatives.
