# Correction: Towards a Better Detection of Horizontally Transferred Genes by Combining Unusual Properties Effectively

**DOI:** 10.1371/journal.pone.0211805

**Published:** 2019-01-31

**Authors:** Dapeng Xiong, Fen Xiao, Li Liu, Kai Hu, Yanping Tan, Shunmin He, Xieping Gao

After publication of this article [1], it was raised that the link of the HGT-DB database (http://genomes.urv.cat/HGT-DB) that the authors cited in the article has become unavailable. The authors contacted the author of the HGT-DB database who notes that the database is now unavailable and the HGT predictions are obsolete.

Using the last version of the HGT-DB, authors implemented the method described in the article [1]. The last version of the HGT-DB database contains outdated genome annotations and HGT predictions, however authors confirm that their proposed method remains feasible and reliable. All the data, codes and details can be downloaded from https://github.com/gpanda1/for_hgt.

In addition, authors erroneously referred to definitions of Type I error and Type II error in the “Evaluation Criteria” section of the Materials and Methods. Please see the complete “Evaluation Criteria” section here with the correct definitions of Type I and Type II errors:

In this research, we used detection rate Recall as our primary evaluation criteria as the same with that in the paper published by Azad et al. [11]. In addition, Mean error was also used as the evaluation criteria to sufficiently evaluate the performance of Hgtident. They are defined as follows,
  $$Recall=\frac{TP}{TP+FN}x\;100,$$
  $$Mean\;error=\frac{Type\;I\;error+Type\;II\;error}{2}x\;100,$$
  Where $Type\;I\;error=\frac{FP}{TN+FP},\;Type\;II\;error=\frac{FN}{TP+FN}$, and TP refers to true positives, FN refers to false negatives, TN refers to true negatives and FP refers to false positives.

These definitions do not affect the results, because the mean of Type I error and Type II error have been used as evaluation measures.
